# Interleukin‐33 increases the sensitivity of multiple myeloma cells to the proteasome inhibitor bortezomib through reactive oxygen species‐mediated inhibition of nuclear factor kappa‐B signal and stemness properties

**DOI:** 10.1002/mco2.562

**Published:** 2024-05-09

**Authors:** Ruonan Shao, Shuang Liu, Wenjian Liu, Cailu Song, Lingrui Liu, Lewei Zhu, Fu Peng, Yue Lu, Hailin Tang

**Affiliations:** ^1^ State Key Laboratory of Oncology in South China, Guangdong Provincial Clinical Research Center for Cancer Sun Yat‐sen University Cancer Center Guangzhou PR China; ^2^ Department of Oncology the Third Affiliated Hospital of Soochow University Changzhou Jiangsu PR China; ^3^ The First People's Hospital of Foshan Foshan PR China; ^4^ West China School of Pharmacy Sichuan University Chengdu PR China

**Keywords:** bortezomib, IL‐33, multiple myeloma, NF‐κB, ROS, stemness

## Abstract

The proteasome inhibitor bortezomib (BTZ) is the first‐line therapy for multiple myeloma (MM). BTZ resistance largely limits its clinical application in MM. Interleukin‐33 (IL‐33) exerts antitumor effects through various mechanisms, including enhancing antitumor immunity and promoting the apoptosis of cancer cells. Here, the synergistic anti‐MM effect of IL‐33 and BTZ was verified, and the underlying mechanisms were elucidated. Bioinformatic analysis indicated that IL‐33 expression levels were downregulated in MM, and that BTZ‐treated MM patients with high IL‐33 levels had better prognosis than those with low IL‐33 levels. Moreover, the patients with high IL‐33 levels had a better treatment response to BTZ. Further immune analysis suggested that IL‐33 can enhance the anti‐MM immunity. IL‐33 and BTZ synergistically inhibited proliferation and induced apoptosis of MM cells, which was mediated by the excessive accumulation of cellular reactive oxygen species (ROS). Furthermore, increased ROS hindered the nuclear translocation of NF‐κB‐p65, thereby decreasing the transcription of target stemness‐related genes (*SOX2*, *MYC*, and *OCT3/4*). These effects induced by the combination therapy could be reversed by eliminating ROS by *N*‐acetylcysteine. In conclusion, our results indicated that IL‐33 enhanced the sensitivity of MM to BTZ through ROS‐mediated inhibition of nuclear factor kappa‐B (NF‐κB) signal and stemness properties.

## Graphical abstract

Bortezomib and IL‐33 synergistically resulted in excessive accumulation of cellular reactive oxygen species (ROS) and depletion of glutathione (GSH), which in turn damaged DNA and ultimately triggered cell death. In addition, ROS overproduction hindered the nuclear translocation of NF‐κB‐p65, thereby decreasing the transcription levels of target stemness‐related genes (*SOX2*, *MYC*, and *OCT3/4*). ROS elimination by *N*‐acetylcysteine intervention can reverse the inhibition of nuclear factor kappa‐B (NF‐κB) activity and its transcriptional targets.

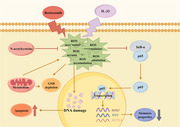

## 1 INTRODUCTION

Multiple myeloma (MM) is a hematological malignancy manifested by the excessive accumulation and infiltration of nonfunctional plasma cells in the bone marrow.^1^, ^2^ It is the second most common hematological malignancy after non‐Hodgkin's lymphoma, and it mainly occurs in elderly men over 65 years of age.^3^ In recent years, the wide administration of new drugs, particularly the proteasome inhibitor bortezomib (BTZ), has greatly prolonged the survival time of MM patients.^4^, ^5^, ^6^ However, MM treatment still faces the challenge of intrinsic and acquired BTZ resistance, and MM remains incurable.^4^, ^5^, ^6^ MM stem cell‐like cells (MMSCs) are considered to be the primary underlying source of BTZ resistance.^7^, ^8^ MMSCs have been identified in MM and confer MM cells with the abilities of self‐renewal and multidrug resistance.^8^, ^9^ Side population, sphere formation capacity, and cancer stem cell (CSC) core genes (*SOX2*, *MYC*, *OCT3/4*, and *NANOG*) are crucial markers of MMSCs.^8^, ^9^, ^10^ The nuclear factor kappa‐B (NF‐κB) signaling pathway plays a vital role in the maintenance of CSCs and drug resistance by modulating stemness‐related genes in cancers.^8^ NF‐κB signal is markedly activated in MM and is crucial for MM progression and BTZ resistance.^8^ Activation of NF‐κB signal results in the nuclear translocation of NF‐κB‐p65, which subsequently regulates the transcription of target genes.^11^ Blocking NF‐κB‐p65 activity and reducing stemness properties can enhance the sensitivity of MM to BTZ.^8^, ^12^ Consequently, targeting NF‐κB signal and MMSCs may be promising therapeutic strategies for improving MM survival outcome.

Reactive oxygen species (ROS) are two electron reduction products of oxygen generated in oxidative metabolism and implicated in various human diseases.^13^ Glutathione (GSH) is the major antioxidant in antioxidative system that can scavenge ROS.^14^, ^15^ Excessive accumulation of cellular ROS depletes GSH, which consequently damages DNA, proteins, and lipids, and ultimately induces cell death.^14^, ^15^ High levels of cellular ROS have antitumor effects by triggering various forms of cell death, including but not limited to apoptosis, autophagy, necroptosis, and ferroptosis.^14^, ^16^ ROS generation served as an important mechanism of BTZ‐induced cytotoxicity.^17^ In contrast to cancer cells, CSCs have lower ROS levels and higher GSH levels physiologically, which are associated with chemotherapy resistance.^18^ Many studies have shown that elevated ROS can reduce cancer stemness, drug resistance, radiation resistance, and tumor progression, as well as inhibit NF‐κB activity.^18^, ^19^, ^20^, ^21^ Increasing ROS production enhances the sensitivity of MM cells to BTZ.^22^, ^23^ Interestingly, there seems to exist a connection between BTZ, ROS, NF‐κB signal, and CSCs.

Interleukin‐33 (IL‐33) is a member of the IL‐1 cytokine family. It exerts vital biological functions in various inflammatory diseases and cancers by interacting with and activating its receptor ST2 upon secretion.^24^ IL‐33 can act as either an oncogene or a tumor suppressor in different tumor types. The majority of studies have investigated the antitumor effects of IL‐33 by enhancing antitumor immunity.^25^ Several studies have shown that IL‐33 also has significant antitumor efficacy by suppressing proliferation and inducing apoptosis of cancer cells.^26^, ^27^ However, its biological role in MM remains unclear.

A previous study revealed that IL‐33/ST2 stimulates the generation of neutrophil‐derived ROS.^28^ Inspired by the connection between “BTZ, ROS, NF‐κB signal, and CSCs” and the crucial role of IL‐33 in ROS generation, we hypothesized that IL‐33 could enhance the sensitivity of MM cells to BTZ through ROS‐mediated inhibition of NF‐κB signal and stemness properties. Here, the synergistic anti‐MM effect of IL‐33 and BTZ was verified, and the underlying mechanisms were elucidated.

## 2 RESULTS

### 2.1 Correlation of IL‐33 levels with prognosis and clinicopathological characteristics in MM patients

**FIGURE 1:**
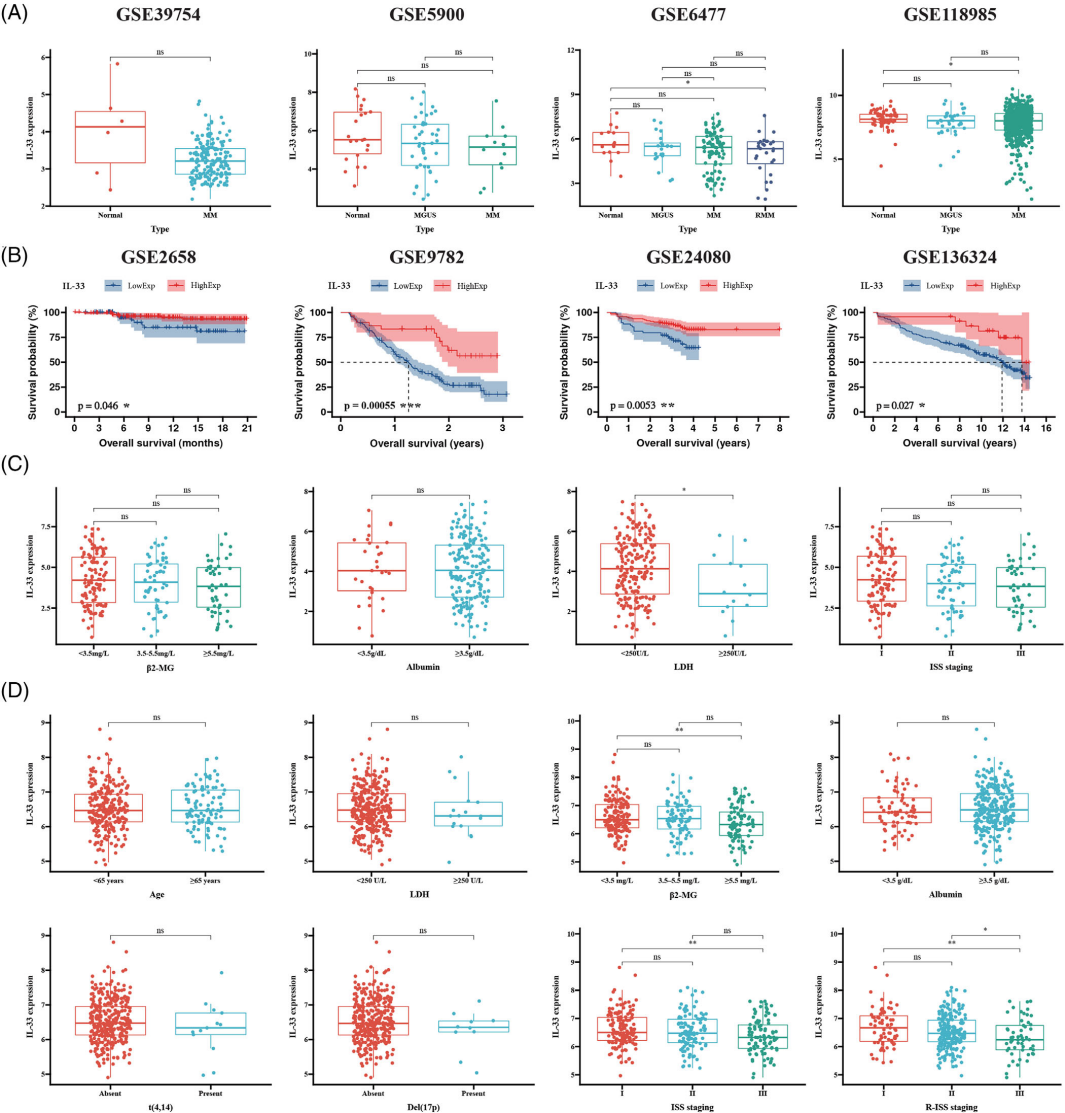
Expression signatures and prognostic significance of interleukin‐33 (IL‐33) in multiple myeloma (MM). (**A**) Expression signatures of IL‐33 in four cohorts. (**B**) Kaplan–Meier analysis of overall survival for bortezomib (BTZ)‐treated MM patients with high or low IL‐33 expression in four cohorts. (**C**) The correlation of IL‐33 expression levels with β2‐microglobulin (β2‐MG), albumin, lactate dehydrogenase (LDH), and ISS staging in the GSE24080 cohort. (**D**) The correlation of IL‐33 expression levels with age, LDH, β2‐MG, albumin, t(4,14), del(17p), ISS, and R‐ISS staging in the GSE136324 cohort (*p < 0.05, **p < 0.01, and ***p < 0.001).

To better understand the clinical significance of IL‐33 in MM patients, we validated the IL‐33 expression profiles in four cohorts. According to our results, IL‐33 expression levels were reduced in monoclonal gammopathy of undetermined significance, MM, and relapsed MM patients, relative to normal contributors (*p* < 0.05; Figure 1A). By performing Kaplan–Meier analysis, we revealed that IL‐33 was a favorable prognostic factor for overall survival in BTZ‐treated MM patients from four cohorts (*p* < 0.05; Figure 1B). Furthermore, the low IL‐33 expression patients had increased lactate dehydrogenase levels in the GSE24080 cohort (*p* < 0.05; Figure 1C), as well as higher β2‐microglobulin levels and worse international staging system (ISS) and revised international staging system (R‐ISS) staging in the GSE136324 cohort (*p* < 0.05; Figure 1D). No clear correlation between IL‐33 level and albumin, age, t(4,14), and del (17p) was observed (Figure 1D). Collectively, high IL‐33 levels were associated with better outcome and less severe clinical characteristics in MM patients.

### 2.2 Recognition of the IL‐33‐related immune features and checkpoints in the GSE136324 cohort

**FIGURE 2:**
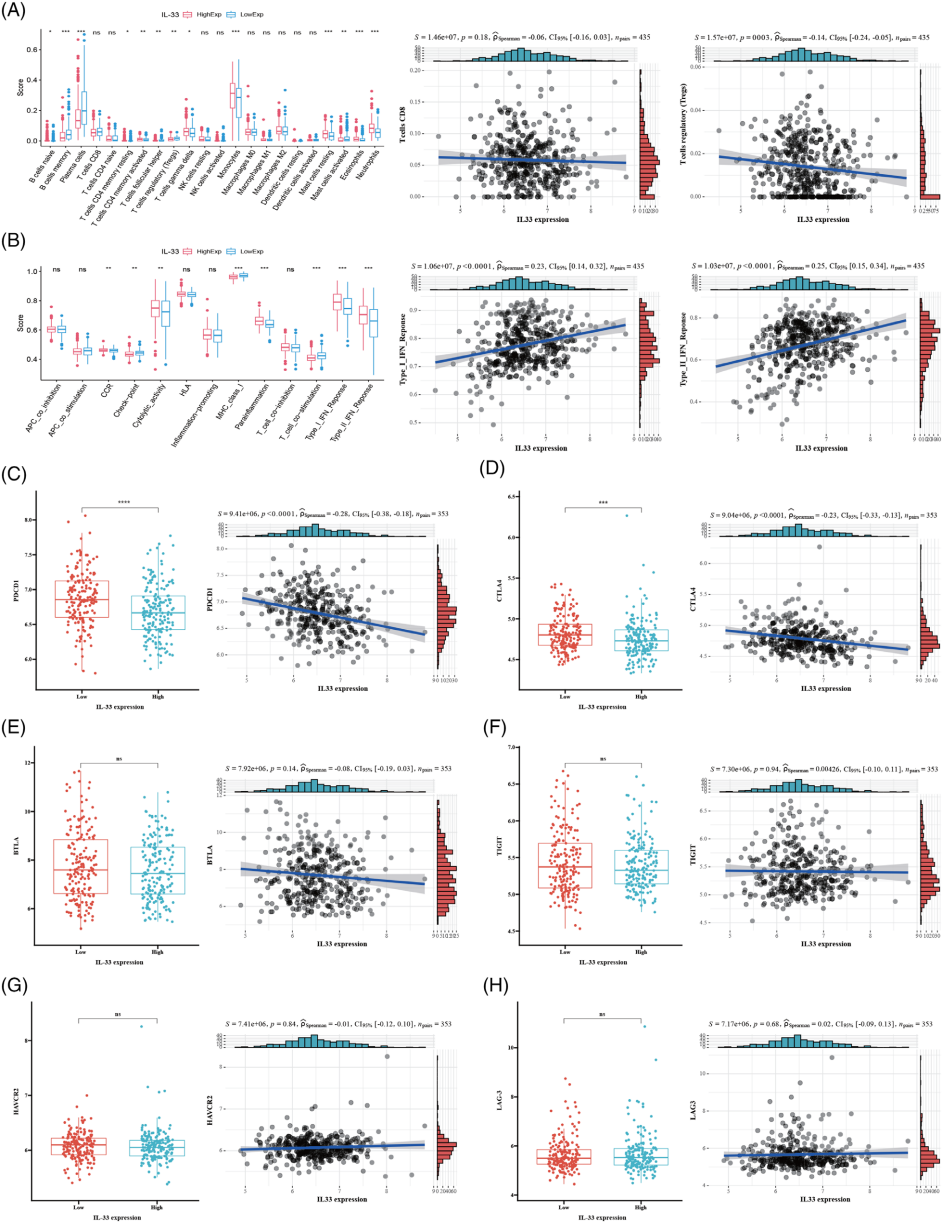
Investigations of interleukin‐33 (IL‐33)‐related immune features and checkpoints in the GSE136324 cohort. (**A**) The correlation of IL‐33 expression levels with the score of 22 immune cells. (**B**) The correlation of IL‐33 expression levels with the score of 13 immune‐related functions. (**C–H**) Relationships between IL‐33 and expression levels of PDCD1 (**C**), CTLA4 (**D**), BTLA (**E**), TIGIT (**F**), HAVCR2 (**G**), and LAG‐3 (**H**) in the GSE136324 cohort. APC, antigen‐presenting cell; CCR, cytokine–cytokine receptor; HLA, human leukocyte antigen; MHC, major histocompatibility complex; NK, natural killer (*p < 0.05, **p <  0.01, ***p <  0.001, and ****p <  0.0001).

Considering that IL‐33 plays crucial roles in type 2 immunity, inflammation, and viral infection, we further explored the effects of IL‐33 on immune infiltration based on public database. According to our results, the activated CD4 memory T cells, gamma and delta T cells, monocytes and neutrophils were significantly enriched in IL‐33‐high group. While the T cells regulatory (Tregs) score was decreased in the high IL‐33 expression group (Figure 2A). Furthermore, the cytolytic activity and interferon (IFN) response scores were notably elevated in the high IL‐33 expression group (Figure 2B). These results indicated that IL‐33 was positively correlated with antitumor immunocyte infiltration in MM.

Next, we examined the expression levels of eight potential immune checkpoints in the low‐ and high‐IL‐33 groups in the GSE136324 cohort. PDCD1 (PD‐1) and CTLA4 expression levels were remarkably higher in the low IL‐33 group, indicating that MM patients with lower IL‐33 expression levels might have a better therapeutic outcome to therapies targeting the checkpoints above (Figure 2C,D). Other immune checkpoints (BTLA, TIGHT, HAVCR2, and LAG3) showed no differential expression between two groups (Figure 2E–H).

### 2.3 IL‐33 enhanced BTZ‐mediated anti‐MM efficacy

**FIGURE 3:**
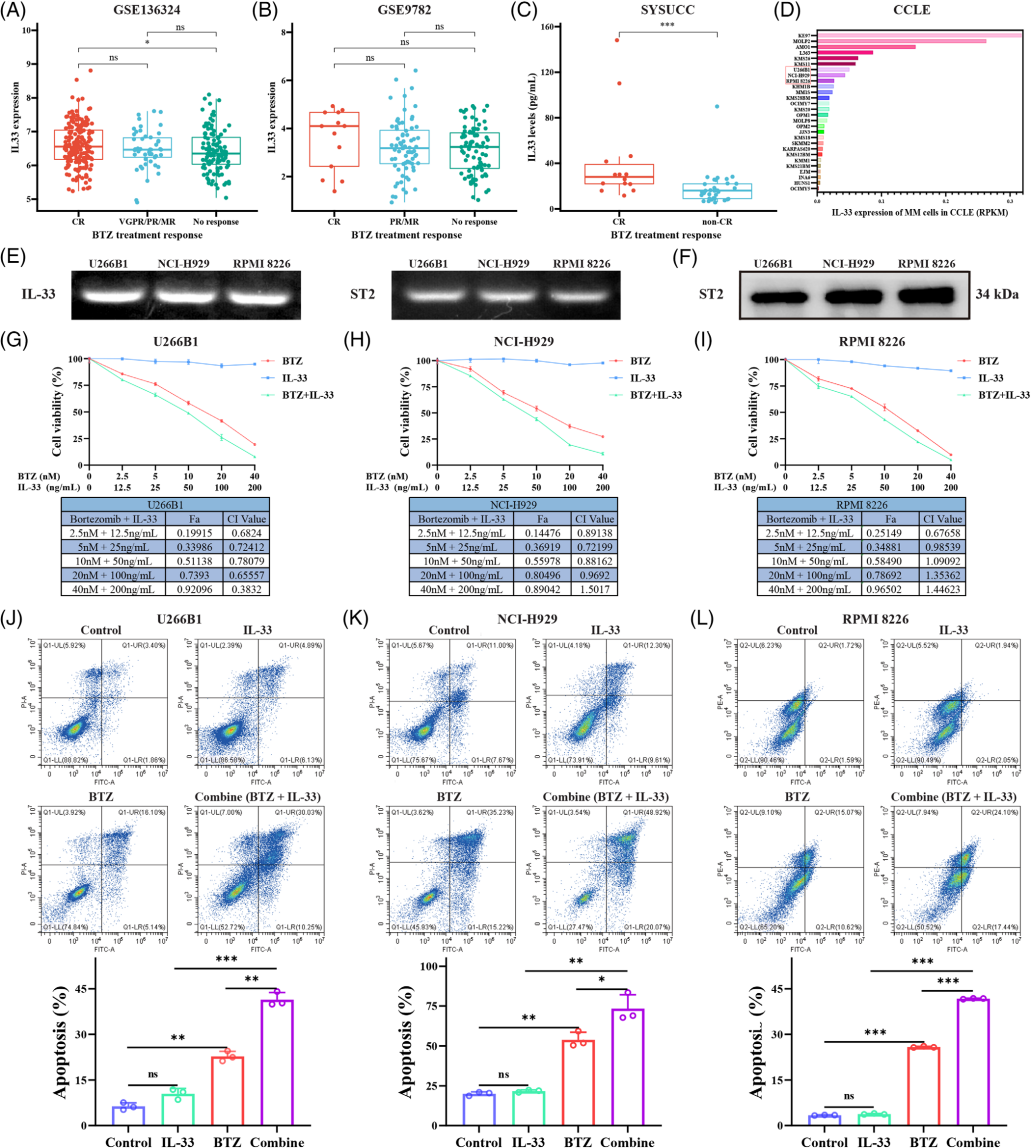
Interleukin‐33 (IL‐33) enhanced bortezomib (BTZ)‐mediated antimultiple myeloma (MM) efficacy. (**A, B**) The correlation of IL‐33 expression levels with BTZ treatment response in two cohorts. CR, complete remission; MR, minimal response; PR, partial response; VGPR, very good partial response. (**C**) The correlation of plasma IL‐33 levels with BTZ treatment response in Sun Yat‐sen University Cancer Center (SYSUCC) cohort. (**D**) IL‐33 expression levels of MM cell lines in CCLE. (**E**) Quantitative real‐time polymerase chain reaction (qRT‐PCR) analysis of the expression of IL‐33 and ST2 in MM cells. (**F**) Western blot (WB) analysis of the expression of ST2 in MM cells. (**G–I**) MM cells were treated with various concentrations of IL‐33 (0, 12.5, 25, 50, 100, 200 ng/mL) and/or BTZ (0, 2.5, 5, 10, 20, 40 nM) for 48 h, and the cell viability was determined via Cell Counting Kit‐8 (CCK‐8) assay. Combination index (CI) values of IL‐33 and BTZ were summarized in tables. (**J–L**) Apoptosis was assessed via flow cytometry in MM cells treated with IL‐33 (100 ng/mL) and/or BTZ (5 nM) for 48 h. All experiments were performed in triplicate and data were presented as the mean ± SD (*p < 0.05, **p < 0.01, and ***p < 0.001).

To further explore the role of IL‐33 in MM, we investigated the relationship between IL‐33 level and BTZ treatment response in MM patients. We found that the patients who achieved complete remission (CR) after BTZ treatment exhibited higher IL‐33 expression levels, relative to patients who failed to respond to BTZ treatment in the GSE136324 cohort (*p* < 0.05; Figure 3A,B). Then we validated this conclusion in Sun Yat‐sen University Cancer Center (SYSUCC) cohort. We measured plasma IL‐33 levels in 50 BTZ‐treated MM patients, comprising 14 CR and 36 non‐CR patients. The CR patients exhibited obviously higher plasma IL‐33 levels compared to non‐CR patients (Figure 3C). These results indicated that the IL‐33 high expression patients had better BTZ treatment response. We hypothesized that IL‐33 can enhance the sensitivity of MM to BTZ.

We investigated the IL‐33 expression levels of MM cell lines in Cancer cell Line Encyclopedia (CCLE) database (Figure 3D). Three MM cell lines with medium IL‐33 expression level, including U266B1, NCI‐H929, and RPMI 8226 cells, were employed for subsequent experiments. We validated the IL‐33 and ST2 expression of U266B1, NCI‐H929, and RPMI 8226 cells by quantitative real‐time polymerase chain reaction (qRT‐PCR) assay (Figure 3E). The ST2 protein levels of U266B1, NCI‐H929, and RPMI 8226 cells were further confirmed by Western blot (WB) assay (Figure 3F).

We performed proliferation study to evaluate the effect of IL‐33 in vitro. MM cells were treated with different doses of IL‐33 and BTZ either alone or in combination for 48 h before performing Cell Counting Kit‐8 (CCK‐8) assay. We observed that IL‐33 monotherapy did not markedly suppress the growth of MM cells, whereas IL‐33 intervention remarkably enhanced the antiproliferative activity of BTZ in MM cells. BTZ and IL‐33 synergistically inhibited MM cells proliferation at combination index (CI) values <1 (Figure 3G–I). Then, we employed flow cytometry to assess MM cells apoptosis after single or combined treatment with IL‐33 and BTZ. We observed that IL‐33 monotherapy did not potently induce MM cell apoptosis, while IL‐33 treatment markedly increased the cytotoxicity of BTZ in MM cells (Figure 3J–L). Overall, we revealed that IL‐33 could enhance the antitumor effect of BTZ in vitro.

### 2.4 IL‐33 attenuated the NF‐κB signal and stemness properties of MM cells in the presence of BTZ

**FIGURE 4:**
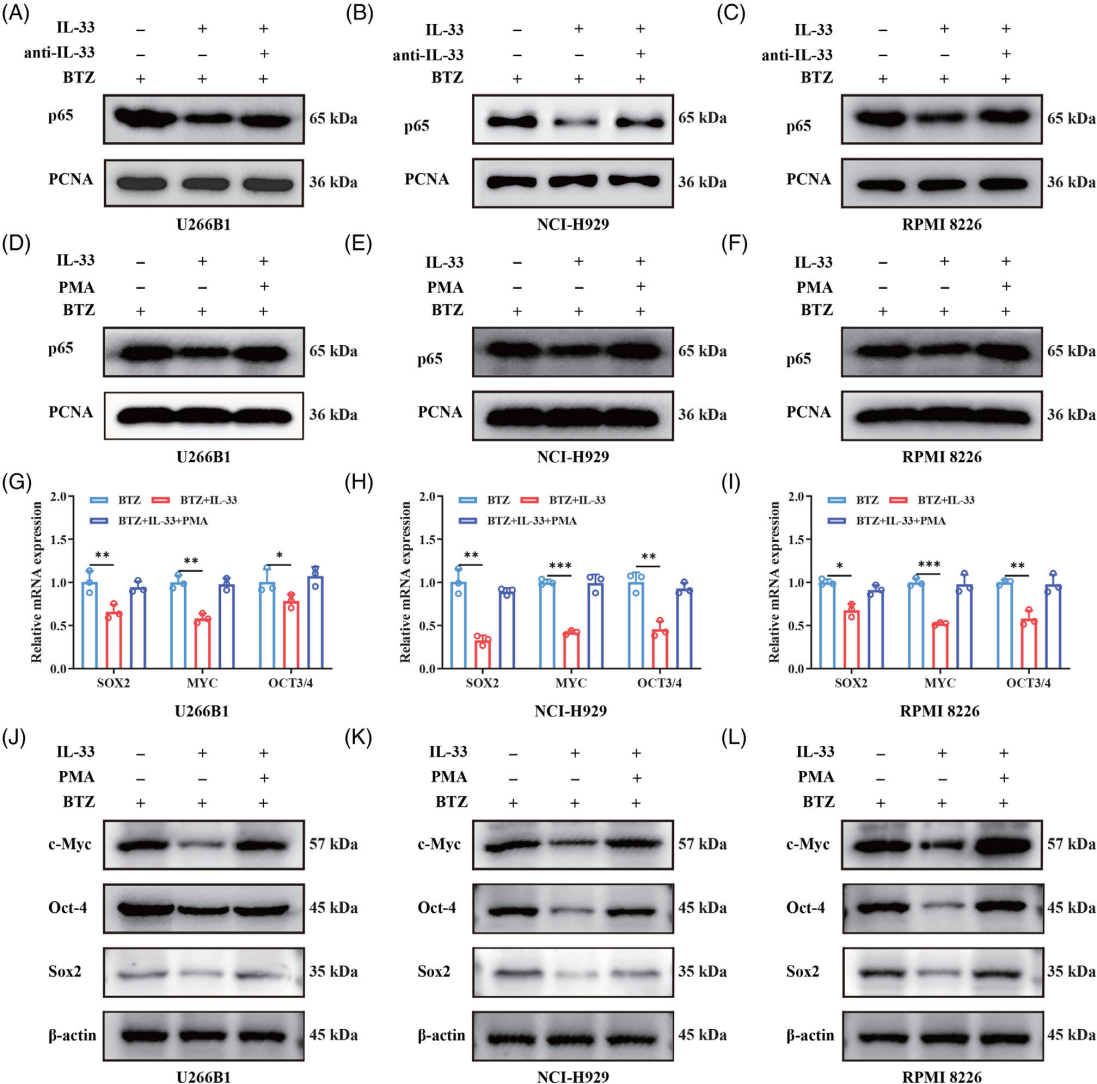
Interleukin‐33 (IL‐33) attenuated the nuclear factor kappa‐B (NF‐κB) signal and stemness properties of multiple myeloma (MM) cells in the presence of bortezomib (BTZ). MM cells were incubated with different combination of compounds, including BTZ (5 nM), IL‐33 (100 ng/mL), anti‐IL‐33 (1 ug/mL), and phorbol 12‐myristate 13‐acetate (PMA; 50 nM), for 48 h, and then the Western blot (WB) and quantitative real‐time polymerase chain reaction (qRT‐PCR) assay were conducted. (**A–F**) The nuclear protein levels of NF‐κB p65 were accessed by WB assay, proliferating cell nuclear antigen (PCNA) as control. (**G–I**) The transcription levels of *SOX2*, *MYC*, and *OCT3/4* were measured by qRT‐PCR assay. (**J–L**) The protein levels of c‐Myc, Oct‐4, and Sox2 were accessed by WB assay, β‐actin as control. All experiments were performed in triplicate and data were presented as the mean ± SD (*p < 0.05, **p < 0.01, and ***p < 0.001).

We next interrogate the underlying mechanism through which IL‐33 enhanced the antitumor effect of BTZ. Previous studies demonstrated that constitutive NF‐κB activation and MM stem cells are believed to be the main causes of BTZ resistance. Thus, we investigated the nuclear translocation of NF‐κB‐p65 subunit, and the expression levels of CSC core genes (*SOX2*, *MYC*, and *OCT3/4*) in MM cells after exposure to specific agents. We observed that IL‐33 decreased the nuclear NF‐κB p65 protein levels of MM cells in the presence of BTZ, and this phenomenon could be reversed by the intervention of anti‐IL33 or NF‐κB activator phorbol 12‐myristate 13‐acetate (PMA; Figure 4A–F). Further researches indicated that IL‐33 attenuated the transcription levels of *SOX2*, *MYC*, and *OCT3/4* of MM cells in the presence of BTZ, which could be reversed by PMA (Figure 4G–I). As expected, the protein levels of c‐Myc, Oct‐4, and Sox2 were significantly downregulated after the addition of IL‐33 in the presence of BTZ, and this results could also be reversed by PMA (Figure 4J–L). In summary, IL‐33 may enhanced the antitumor effect of BTZ through attenuating the NF‐κB signal and stemness properties of MM cells.

### 2.5 IL‐33 enhanced BTZ‐induced cytotoxicity through ROS‐dependent manners in MM

**FIGURE 5:**
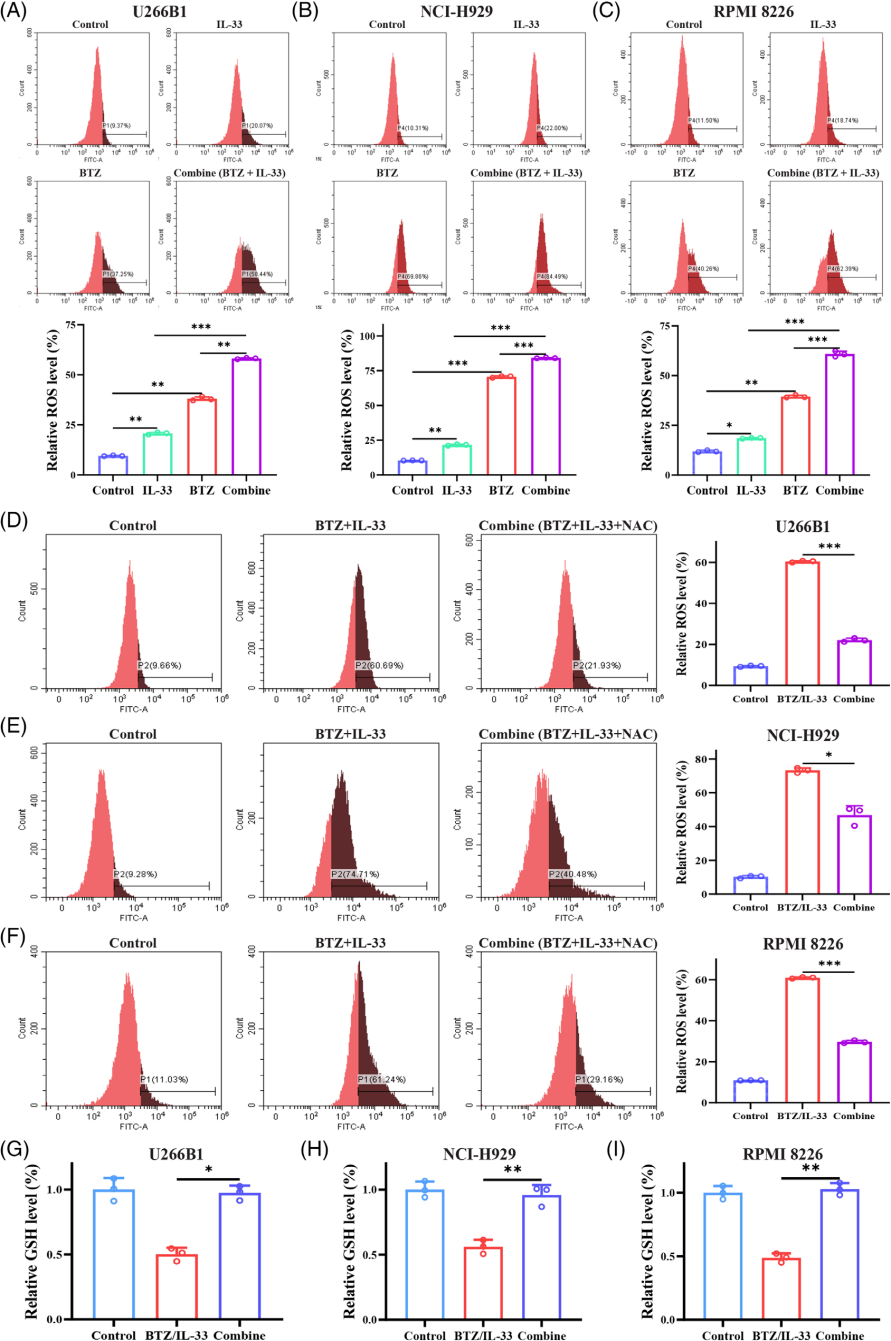
Interleukin‐33 (IL‐33) and bortezomib (BTZ) synergistically resulted in excessive accumulation of cellular reactive oxygen species (ROS) in multiple myeloma (MM) cells. (**A–C**) ROS levels were assessed via flow cytometry in MM cells treated with IL‐33 (100 ng/mL) and/or BTZ (5 nM) for 48 h. (**D–I**) MM cells were incubated with IL‐33 (100 ng/mL) and BTZ (5 nM), with or without *N*‐acetylcysteine (NAC) (10 mM) addition for 48 h, and then the ROS levels (**D–F**) and glutathione (GSH) levels (**G–I**) were assessed. All experiments were performed in triplicate and data were presented as the mean ± SD (*p < 0.05, **p < 0.01, and ***p < 0.001).

**FIGURE 6:**
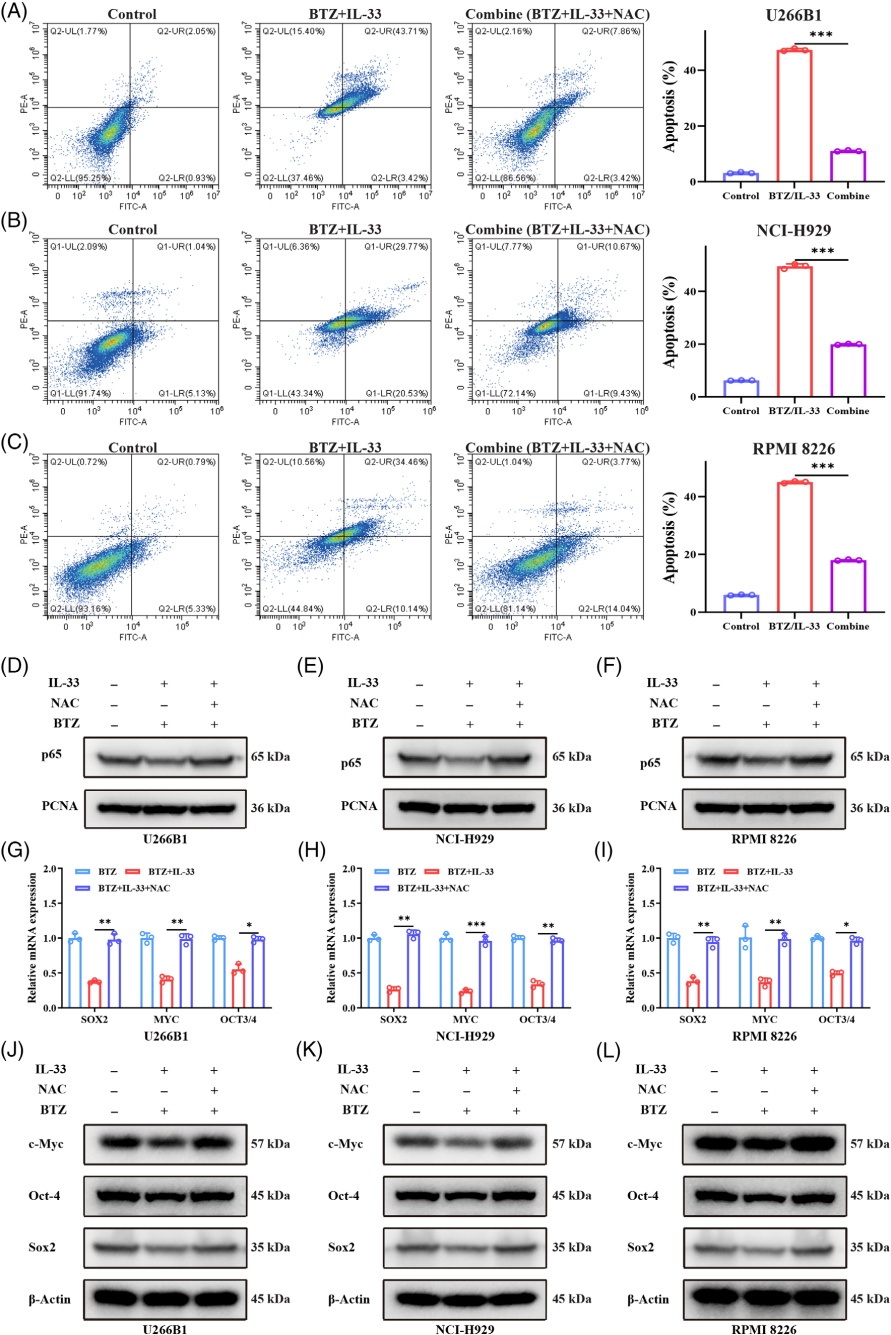
Interleukin‐33 (IL‐33) attenuated the nuclear factor kappa‐B (NF‐κB) signal and stemness properties of multiple myeloma (MM) cells in the presence of bortezomib (BTZ) via inducing reactive oxygen species (ROS) production. MM cells were incubated with IL‐33 (100 ng/mL) and BTZ (5 nM), with or without *N*‐acetylcysteine (NAC) (10 mM) addition for 48 h, and then the flow cytometry, Western blot (WB), and quantitative real‐time polymerase chain reaction (qRT‐PCR) assay were conducted. (**A–C**) The apoptosis was assessed via flow cytometry. (**D–F**) The nuclear protein levels of NF‐κB p65 were accessed through WB assay, PCNA as control. (**G–I**) The transcription levels of *SOX2*, *MYC*, and *OCT3/4* were measured by qRT‐PCR assay. (**J–L**) The protein levels of c‐Myc, Oct‐4, and Sox2 were accessed through WB assay, β‐actin as control. All experiments were performed in triplicate and data were presented as the mean ± SD (*p < 0.05, **p < 0.01, and ***p < 0.001).

ROS generation served as an important mechanism of BTZ‐induced cytotoxicity. Therefore, we measured the ROS levels of MM cells after mono‐ or combination therapies with IL‐33 and BTZ. Our results showed that IL‐33 or BTZ monotherapy could markedly increase the production of cellular ROS. Furthermore, IL‐33 and BTZ synergistically resulted in excessive accumulation of ROS (Figure 5A–C). The excessive ROS accumulation in MM cells could be reversed by *N*‐acetylcysteine (NAC) addition (Figure 5D–F). Moreover, combination therapy with IL‐33 and BTZ led to a remarkable decrease of GSH in MM cells which could be restored by NAC intervention (Figure 5G–I). More importantly, ROS elimination by NAC could reduce the apoptosis of MM cell caused by combination therapy (Figure 6A–C). Our result showed that IL‐33 enhanced the effect of BTZ partially through ROS‐dependent manners.

### 2.6 IL‐33 attenuated the NF‐κB signal and stemness properties of MM cells in the presence of BTZ via inducing ROS production

Previous studies have shown that ROS overproduction could inhibit NF‐κB activity and attenuate cancer stemness. We then hypothesized that IL‐33 attenuated the NF‐κB signal and stemness properties of MM cells in the presence of BTZ via inducing ROS production. Further experiments were performed to verify our hypothesis. We found that NAC intervention could reverse the inhibition of NF‐κB activity caused by the combination therapy with IL‐33 and BTZ (Figure 6D–F). In addition, the transcription levels of *SOX2*, *MYC*, and *OCT3/4* were significantly downregulated by the combination therapy and this phenomenon could be restored by NAC (Figure 6G–I). Similarly, the protein levels of c‐Myc, Oct‐4, and Sox2 decreased by the combination therapy could also be recovered by NAC (Figure 6J–L). Taken together, IL‐33 inhibited the NF‐κB signal and stemness properties of MM cells via inducing ROS production.

### 2.7 IL‐33 potentiated the inhibitory effect of BTZ on MM progression in xenograft models

**FIGURE 7:**
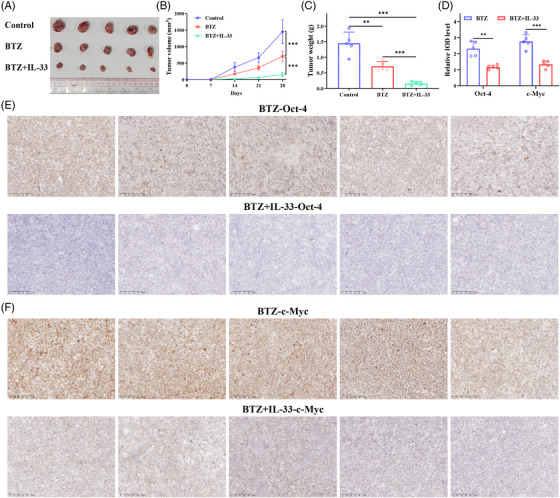
Interleukin‐33 (IL‐33) potentiated the inhibitory effect of bortezomib (BTZ) on multiple myeloma (MM) progression in U226B1 cell xenograft models. (**A**) Representative images of tumors removed from the mice. (**B**) Tumor volumes in three groups were observed on the indicated days. (**C**) Tumor weights in three groups were shown after removed. (**D**) Relative integrated optical density (IOD) levels of Oct‐4 and c‐Myc in MM xenografts of two groups. (**E, F**) Immunohistochemistry (IHC) staining images (Oct‐4 and c‐Myc) of U226B1 cell xenograft tumor tissues. Data were presented as the mean ± SD (*p < 0.05, **p < 0.01, and ***p < 0.001).

To further confirm the role of IL‐33 in BTZ‐mediated anti‐MM activity in vivo, we constructed U226B1 cell xenograft mouse model to evaluate antitumor efficacy of IL‐33 and BTZ through mono‐ or combination therapies. Our results indicated that IL‐33 treatment potently enhanced the MM growth inhibition induced by BTZ monotherapy (Figure 7A–C). The expression levels of Oct‐4 and c‐Myc in MM xenografts were also estimated by immunohistochemistry (IHC). Consistent with the in vitro findings, combination therapy significantly decreased the expression of Oct‐4 and c‐Myc in MM cells compared with BTZ monotherapy treatment (Figure 7D–F). In summary, IL‐33 enhanced the effect of BTZ as well as inhibiting the stemness properties of MM cells in vivo.

## 3 DISCUSSION

BTZ is the first therapeutic proteasome inhibitor employed in MM management.^3^ And BTZ usage has significantly improved the therapeutic effect of MM.^4^, ^6^ However, BTZ resistance is quite prevalent, and the overall survival of BTZ‐resistant patients is extremely poor.^8^ Thus, elucidating the mechanisms of BTZ resistance is essential for improving the survival of MM patients. BTZ monotherapy induces an increase in CSC properties, and this is an important mechanism behind BTZ resistance in MM.^29^, ^30^ Several studies have indicated that bioactive compounds can overcome BTZ resistance by targeting CSCs through various signaling pathways, such as NF‐κB and transforming growth factor beta (TGF‐β), and the modulation of ROS.^8^, ^12^, ^22^, ^31^ Emerging evidences suggested that IL‐33 exerts antitumor effects through various mechanisms, including enhancing antitumor immunity, inhibiting proliferation, and promoting apoptosis of cancer cells.^25^, ^26^, ^27^, ^32^, ^33^ A previous study showed that low plasma IL‐33 levels are related to advanced staging of MM,^34^ indicating that IL‐33 may be a favorable prognostic indicator of MM. Nevertheless, there is limited information regarding the biological role of IL‐33 in MM. Thus, we investigated the role of IL‐33 in MM using bioinformatic analysis. We revealed that IL‐33 expression levels were downregulated in MM relative to healthy donors, and that BTZ‐treated MM patients with high IL‐33 levels had better prognosis than those with low IL‐33 levels. Moreover, we discovered that patients with high IL‐33 levels had a better treatment response to BTZ. These results indicated that IL‐33 was a good prognostic predictor for BTZ‐treated MM patients and IL‐33 could improve the treatment response to BTZ. Then, we performed immune analysis, and in vitro and in vivo experiments to further explore the underlying mechanisms.

We first investigated the relationship between IL‐33 expression levels and immune activities and checkpoints. We demonstrated that patients with high IL‐33 levels exhibited higher IFN levels and lower Treg scores. Moreover, immune checkpoints PDCD1 (PD‐1) and CTLA4 were significantly upregulated in patients with low IL‐33 levels. IFN exerts its antitumor effects by enhancing the antitumor immunity.^35^, ^36^, ^37^ Many studies have reported that a high proportion of Tregs infiltrates in the tumor microenvironment and Tregs modulate immune escape by suppressing the antitumor immunity.^38^, ^39^, ^40^ Also abundant Tregs infiltration is associated with poor prognosis of hematological malignancies.^38^, ^39^, ^40^ PD‐1 and CTLA4 play critical roles in tumor immune escape by inhibiting T cell activation.^40^, ^41^ These findings suggested that IL‐33 could enhance anti‐MM immunity to improve the prognosis of MM.

In vitro experiments demonstrated that IL‐33 alone did not have obvious cytotoxicity to MM cells, whereas IL‐33 combined with BTZ could synergistically inhibit proliferation and promote apoptosis of MM cells, suggesting that IL‐33 could enhance BTZ‐mediated anti‐MM efficacy. NF‐κB activation and MMSCs are main sources of BTZ resistance.^7^, ^8^ As expected, the addition of IL‐33 significantly inhibited the NF‐κB activity and stemness properties of MM cells in the presence of BTZ. ROS generation is an important mechanism of BTZ cytotoxicity and that IL‐33 can also induce the production of cellular ROS.^17^, ^28^ Increased ROS could inhibit NF‐κB activity and reduce cancer stemness properties, which ultimately suppress tumor progression and drug resistance.^18^, ^20^, ^21^, ^42^ We hypothesized that the combination therapy of IL‐33 and BTZ could significantly promote the generation of ROS, which induced MM cell death, in addition, excessive ROS accumulation attenuated the NF‐κB activity, thereby decreasing the stemness properties of MM cells. Just as we predicted, we observed that IL‐33 or BTZ alone could markedly increase the production of ROS, and IL‐33 and BTZ synergistically resulted in excessive accumulation of ROS in MM cells. Similarly, ROS elimination by NAC could attenuate the cytotoxicity caused by the combined treatment of IL‐33 and BTZ in MM cells. Furthermore, the inhibition of NF‐κB activity and stemness properties led by the combination therapy could be reversed by NAC intervention in MM cells. These findings provided sufficient evidences for our hypothesis. Our xenograft models further confirmed our findings. However, there was a limitation. Although nude mice lack most of their immunity due to the loss of thymus, they still retained some humoral immunity. And immune analysis showed that IL‐33 could enhance anti‐MM immunity. So we could not completely rule out that the enhancement of IL‐33 on BTZ‐mediated anti‐MM efficacy was related to the effect of IL‐33 on the microenvironment,^43^, ^44^ severe combined immunodeficient mice may be more suitable for our study.

In conclusion, we revealed that MM has reduced IL‐33 levels, which, in turn, is intricately linked to poor prognosis of BTZ‐treated MM patients. Additionally, IL‐33 high expression patients had better treatment response to BTZ. Further immune analysis suggested that IL‐33 can enhance the anti‐MM immunity. In vitro and in vivo experiments demonstrated that IL‐33 and BTZ synergistically exerted anti‐MM efficacy via stimulating excessive accumulation of ROS, thereby attenuating NF‐κB signal and suppressing stemness properties of MM cells.

## 4 MATERIALS AND METHODS

### 4.1 Patients and clinical samples

The expression and clinical information of eight MM cohorts, including GSE39754, GSE5900, GSE6477, GSE118985, GSE2658, GSE9782, GSE24080, and GSE136324, were retrieved from the Gene Expression Omnibus (GEO) database (http://www.ncbi.nlm.nih.gov/geo/). Samples used in each cohort were summarized in Table S1. IL‐33 expression data of MM cell lines were acquired from the CCLE database (https://portals.broadinstitute.org/ccle). Log2 transformation was conducted to normalize the gene expression profiles. All plasma samples were obtained from the SYSUCC. This investigation received approval from the SYSUCC internal review and ethics boards. In this study, we selected patients treated with BTZ from GSE2658, GSE9782, GSE24080, GSE136324, and SYSUCC cohorts.

### 4.2 Immune analysis

The invading scores of 13 immune‐associated activities were computed using the single sample gene set enrichment analysis. Lastly, the invading scores of 22 immune cells were determined through the ESTIMATE algorithm of “estimate” package.

### 4.3 Reagents and antibodies

IL‐33 was purchased from Novoprotein. BTZ was acquired from XIAN JANSSEN. PMA, a type of NF‐κB activators, was acquired from Sigma. NAC, a type of ROS scavengers, was procured from Selleck. Human IL‐33 neutralizing antibody (anti‐IL‐33) was purchased from R&D Systems. Anti‐β‐actin, anti‐Oct4, anti‐Sox2, anti‐c‐Myc, anti‐NF‐κB‐p65, anti‐PCNA, and corresponding secondary antibody were purchased from cell signaling technology. Anti‐ST2 was acquired from Abcam.

### 4.4 Enzyme‐linked immunosorbent assay (ELISA)

Bone marrow plasma IL‐33 levels were assessed using a sandwich ELISA kit (Arigo). The plasma samples were routinely analyzed by ELISA, following kit protocols.

### 4.5 Cell culture

The MM cell lines, namely, U266B1, NCI‐H929, and RPMI 8226, from the American Type Culture Collection were grown in RPMI‐1640 medium with 10% fetal bovine serum, 100 IU/mL penicillin, and 100 mg/mL streptomycin in a 37°C humid chamber with 5% CO_2_.

### 4.6 RNA extraction and quantitative real‐time polymerase chain reaction

MM cells were treated with specified compound for 48 h and then total RNA was isolated using RNA Quick Purification kit (ESscience). The subsequent cDNA conversion was performed with cDNA Reverse Transcription Kit (TAKARA). The subsequent cDNA amplification was done using TB Green® Premix Ex Taq™ Kit (TAKARA). The primers used in qRT‐PCR are summarized in Table S2. Relative gene expression was computed using the 2^−ΔΔCT^ formula.

### 4.7 Cell viability assay

Cell viability was evaluated through CCK‐8 (Dojindo). The MM cells were seeded (10^4^ cells) in 96‐well plate and subsequently, the cells were treated with specified compound for 48 h, prior to CCK‐8 reagent introduction and subsequent incubation for 2 h. Optical density was determined at 450 nm using microplate reader. The CI of IL‐33 and BTZ was determined by CompuSyn software program. CI < 1.0 reflects synergism.

### 4.8 Glutathione quantification, and flow cytometry‐based detection of cell apoptosis and ROS

Total cellular GSH levels were tested using GSSG/GSH Quantification Kit (Beyotime). MM cells were incubated with specified compound for 48 h, prior to harvest, followed by rinsed in chilled phosphate buffered saline (PBS) three times, then GSH levels were measured according to the manufacturer's instructions.

MM cells were incubated with specified compound for 48 h, prior to harvest, followed by rinsed in chilled PBS three times, then FITC‐Annexin V/PI staining was performed using Apoptotic Quantification Kit (KeyGen). After a 15 min incubation in the dark at ambient temperature, cell apoptosis was assessed and quantified using flow cytometry.

ROS levels were tested by DCFH‐DA probe of Reactive Oxygen Species Assay Kit (Beyotime). MM cells pretreated with specified agents for 48 h were incubated with DCFH‐DA probe for 0.5 h. Then cells were harvested and rinsed in chilled PBS three times. Finally, ROS levels were detected by flow cytometry.

### 4.9 Western blot

MM cells were lysed in radio immunoprecipitation assay lysis buffer (RIPA), prior to centrifugation at 12,000 rpm for 15 min at 4°C. Upon protein quantification, the protein samples were combined with loading buffer, before boiling at 100°C for 10 min. The proteins were then electrophoretically separated on a 10% sodium dodecyl sulfate ‐ polyacrylamide gel electrophoresis (SDS–PAGE), then transferred to polyvinylidene fluoride (PVDF) membranes and blocked in 5% skim milk, before incubating with primary antibody and corresponding secondary antibody. Finally, signals were captured with enhanced chemiluminescence and Bio‐Rad imager. The Nuclear and Cytoplasmic Protein Extraction Kit (Beyotime) was applied for distinguishing the cytoplasm and nuclear protein, and then proteins were collected for WB. β‐Actin were used as the internal control of cellular proteins and PCNA was used as the internal control of nuclear proteins.

### 4.10 MM xenograft model and immunohistochemistry

U266B1 cells (10^7^ cells) were subcutaneously administered into female nude mice, and after 1 week of implantation, the subcutaneous xenografts were easily visible to the naked eye (approximately 2 mm). At this point, the mice were intraperitoneally administered IL‐33 (1 ug) and/or BTZ (0.5 mg/kg) twice a week for 4 weeks. At the end of experiment, the mice were euthanized and the xenografts were harvested for weight measurement and other analyses. Our animal protocols received ethical approval from and were conducted at the SYSUCC.

MM xenografts were fixed in formalin and embedded in paraffin, and then sectioned into 5 μm thickness. After deparaffinization and rehydration, sections were blocked and subjected to IHC staining procedure for antibodies by established protocol. Targeted proteins were visualized using diaminobenzidine as substrate. High‐resolution microphotographs were obtained using an electron microscope. The relative integrated optical density of protein expression levels in the IHC slices was measured with Image J software.

### 4.11 Statistical analysis

Continuous data were evaluated via the Student's *t*‐ or Wilcoxon test. The R software (Version 4.0.4), GraphPad Prism, and SPSS (Version 25.0) were employed for all data analyses. A two‐sided *p* < 0.05 was set as the significance threshold. All experiments were conducted thrice, and the resulting data are provided as the mean ± SD.

## AUTHOR CONTRIBUTIONS

RS, SL, and WL conducted the experiments and data analysis. CS and LL provided assistance in animal experimentation and data collection. FP, YL, and HT designed and supervised the study. RS, LZ, FP, YL, and HT wrote the manuscript. All the authors have read and approved the final manuscript.

## CONFLICT OF INTEREST STATEMENT

The authors declare no conflicts of interest in this work.

## ETHICS STATEMENT

This study was approved by the Ethics Committees of SYSUCC. Bone marrow plasma specimens utilized for ELISA in MM patients were obtained retrospectively from SYSUCC (Approval number: B2021‐120‐01). All animal procedures were approved by Institutional Animal Care and Use Committee of SYSUCC (Approval number: L102012019050Q).

## Supporting information

Supporting Information

## Data Availability

All data and materials are available within the article or from the authors upon reasonable request.
