# Supplementary material for: Interleukin‐33 increases the sensitivity of multiple myeloma cells to the proteasome inhibitor bortezomib through reactive oxygen species‐mediated inhibition of nuclear factor kappa‐B signal and stemness properties
Source: MedComm (2020). 2024 May 9;5(5):e562. doi: 10.1002/mco2.562 (PMC11082532; doi:10.1002/mco2.562)
Supplement: Supplementary file 1 — Supporting Information [file MCO2-5-e562-s001.docx]

**IL-33 Increases the Sensitivity of Multiple Myeloma Cells to the Proteasome Inhibitor Bortezomib through** **ROS-Mediated Inhibition of NF-κB** **Signal and Stemness** **P****roperties**

**Ruonan Shao^1,#^ | Shuang Liu^2,#^ | Wenjian Liu^1,#^ | Cailu Song^1^ | Lingrui Liu^1^ | Lewei Zhu^4^ | Fu Peng^3,*^ | Yue Lu^1,*^ | Hailin Tang^1,*^**

State Key Laboratory of Oncology in South China, Guangdong Provincial Clinical Research Center for Cancer, Sun Yat-sen University Cancer Center, Guangzhou, P. R. China.

Department of Oncology, the Third Affiliated Hospital of Soochow University, Changzhou, Jiangsu, P. R. China.

West China School of Pharmacy, Sichuan University, Chengdu, P. R. China

The First People’s Hospital of Foshan, Foshan, P. R. China.

Ruonan Shao, Shuang Liu and Wenjian Liu contributed equally to this study.

**Correspondence**

Hailin Tang, State Key Laboratory of Oncology in South China, Guangdong Provincial Clinical Research Center for Cancer, Sun Yat-sen University Cancer Center, 651 Dongfeng East Road, Guangzhou, 510060, P. R. China. Emails: [tanghl@sysucc.org.cn](mailto:tanghl@sysucc.org.cn); Yue Lu, State Key Laboratory of Oncology in South China, Guangdong Provincial Clinical Research Center for Cancer, Sun Yat-sen University Cancer Center, 651 Dongfeng East Road, Guangzhou, 510060, P. R. China. Emails: [lvyue@sysucc.org.cn](mailto:tanghl@sysucc.org.cn); Fu Peng, West China School of Pharmacy, Sichuan University, Chengdu, P. R. China. Emails: [pengf@scu.edu.cn](mailto:pengf@scu.edu.cn) ;

| **Table S1.** Samples used in each cohort. | |
| --- | --- |
| GSE39754 | CD138 selected bone marrow plasma cells |
| GSE5900 | CD138 selected bone marrow plasma cells |
| GSE6477 | CD138 selected bone marrow plasma cells |
| GSE2658 | CD138 negative selected bone marrow plasma cells |
| GSE9782 | CD138 negative selected bone marrow plasma cells |
| GSE24080 | CD138 selected bone marrow plasma cells |
| GSE118985 | Whole bone marrow biopsies |
| GSE136324 | Whole bone marrow biopsies subtracting plasma cells |

| **Table S2.** qRT-PCR primers for SOX2, MYC, OCT3/4, GAPDH, IL-33 and ST2. | | |
| --- | --- | --- |
| *SOX2* | Forward Primer | TGGACAGTTACGCGCACAT |
|  | Reverse Primer | CGAGTAGGACATGCTGTAGGT |
| *MYC* | Forward Primer | GTCAAGAGGCGAACACACAAC |
|  | Reverse Primer | TTGGACGGACAGGATGTATGC |
| *OCT3/4* | Forward Primer | CTTGAATCCCGAATGGAAAGGG |
|  | Reverse Primer | GTGTATATCCCAGGGTGATCCTC |
| *GAPDH* | Forward Primer | CTGACTTCAACAGCGACACC |
|  | Reverse Primer | TGCTGTAGCCAAATTCGTTGT |
| *IL-33* | Forward Primer | GTGACGGTGTTGATGGTAAGAT |
|  | Reverse Primer | AGCTCCACAGAGTGTTCCTTG |
| *ST2* | Forward Primer | GAAAACCTAGTTACACCGTGGAT |
|  | Reverse Primer | GCAAACACACGATTTCTTTCCTG |
